# Willingness to Use Mobile Phone Apps for HIV Prevention Among Men Who Have Sex with Men in London: Web-Based Survey

**DOI:** 10.2196/mhealth.8143

**Published:** 2017-10-11

**Authors:** William C Goedel, Jason W Mitchell, Paul Krebs, Dustin T Duncan

**Affiliations:** ^1^ Department of Population Health School of Medicine New York University New York, NY United States; ^2^ Office of Public Health Studies University of Hawaii at Manoa Honolulu, HI United States

**Keywords:** mobile phone apps, mHealth, men who have sex with men (MSM), HIV

## Abstract

**Background:**

Many men who have sex with men (MSM) use apps to connect with and meet other MSM. Given that these apps are often used to arrange sexual encounters, it is possible that apps may be suitable venues for messages and initiatives related to HIV prevention such as those to increase HIV testing rates among this population.

**Objective:**

The purpose of this study was to assess willingness to use a new app for reminders of when to be tested for HIV infection among a sample of MSM in London who use apps to arrange sexual encounters.

**Methods:**

Broadcast advertisements targeted users of a popular social-networking app for MSM in London. Advertisements directed users to a Web-based survey of sexual behaviors and sexual health needs. Willingness to use apps for reminders of when to be tested for HIV was assessed. In addition, participants responded to items assessing recent sexual behaviors, substance use, and demographic characteristics. Exploratory analyses were undertaken to examine differences in willingness to use an app by demographic and behavioral characteristics.

**Results:**

Broadcast advertisements yielded a sample of 169 HIV-negative MSM. Overall, two-thirds (108/169, 63.9%) reported willingness to use an app to remind them when to be tested for HIV. There were no significant differences in willingness to use these apps based on demographic characteristics, but MSM who reported recent binge drinking and recent club drug use more frequently reported willingness to use this app compared to their nonusing counterparts.

**Conclusions:**

MSM in this sample are willing to use a new app for HIV testing reminders. Given the high levels of willingness to use them, these types of apps should be developed, evaluated, and made available for this population.

## Introduction

The human immunodeficiency virus (HIV) epidemic in the United Kingdom impacts gay, bisexual, and other men who have sex with men (MSM) at a disproportionate rate compared to the general population. MSM are estimated to represent 3.3% of the male population in the United Kingdom but represent between 66.7% and 69.0% of all males living with HIV infection as of 2015 and 73.0% of newly diagnosed HIV infections among males in 2015 [1]. It is estimated that between 7.2% and 18.9% of HIV-infected MSM are unaware of their infection status and that 30.0% of newly diagnosed HIV-infected MSM are diagnosed “late” (ie, with a CD4 count of less than 350 cells per cubic millimeter within 3 months of diagnosis) [1]. Regular HIV testing has a number of important benefits for MSM who test positive and who test negative. For MSM who test negative, HIV testing provides the opportunity for risk reduction counseling to encourage behavior change that reduces risk for HIV infection and allows the individual to remain HIV negative [2]. For MSM who test positive, HIV testing improves early awareness of infection status and linkage to care [3]. New interventions to increase HIV testing frequency among MSM in the United Kingdom are needed to further identify new cases of HIV infection and effectively link them to treatment and to reduce engagement in risk behaviors by HIV-uninfected and HIV-infected MSM.

For the past decade, the Internet has been used to deliver and disseminate HIV prevention interventions to diverse populations, including gay, bisexual, and other MSM [4]. Frequently, these interventions have included virtual scenarios and simulations, decision making with virtual characters, and detailed answers or feedback following knowledge tests. For example, Health mPowerment, an intervention based on the Institute of Medicine’s integrated model of behavior theory aimed at increasing HIV and sexually transmitted infection (STI) knowledge among young black MSM, incorporates live chats with outreach workers, quizzes, personalized health and journals of sexual activities, and decision support tools for assessing risk behaviors on a single website [5]. Building on these Internet-based interventions and keeping with the pace of technological developments, mobile phone–based HIV prevention interventions are currently in various stages of development and testing. Many of these mobile phone–based interventions incorporate text messaging delivery and have demonstrated some success. For example, Bourne and colleagues [6] found that MSM who enrolled in a text message–based intervention involving reminders for HIV/STI testing tailored based on participant risk behaviors and ability to return for testing were over 4 times more likely to retest for HIV and other STIs compared to those who did not receive the intervention.

Given the pervasiveness of mobile phones and associated apps [7], it may be best to incorporate and adapt aspects of these previously developed Internet- and text message–based interventions for delivery via apps, but it should be noted that their effectiveness is dependent on their continual use, as many individuals download health-related apps and discontinue using them for a variety of reasons [8]. The use of geosocial-networking apps, those that use Global Positioning System (GPS) technology to facilitate various types of connections between MSM, is common among MSM [9-11]. There is no clear consensus in the extant literature regarding the association between meeting sexual partners online or through apps and increased HIV risk behaviors [12]. It has been hypothesized that these digital venues may facilitate multiple partnerships and other HIV risk behaviors for MSM who already engage in these behaviors rather than act as a catalyst for these behaviors themselves [12]. However, while social and sexual network apps are accepted by MSM for HIV prevention and sexual health messaging [13], some companies who produce these geosocial-networking apps are ambiguous in their willingness to support HIV prevention programs [14].

Therefore, the development of new apps for HIV prevention purposes targeted to those potentially high-risk MSM who already use apps to meet sexual partners may be beneficial, but little is known about MSM willingness to engage with new app-related HIV prevention services. As such, the purpose of this study was to examine the willingness to use apps for HIV prevention purposes among a sample of MSM in London recruited from a popular geosocial-networking app used by MSM to meet sexual partners. In exploratory analyses, we examined differences in willingness to use these apps by demographic characteristics and recent sexual and substance use behaviors. This study specifically focused on London because it is an urban epicenter with a high prevalence of HIV among MSM [15] and a high number of MSM who use apps to meet sexual partners [16].

## Methods

### Sample Recruitment

Broadcast advertisements were placed on a popular geosocial-networking app commonly used by MSM to meet sexual partners in January 2016. These broadcasts were targeted specifically to the London metropolitan area. Consistent with recent research among MSM who use apps to meet sexual partners [9], users were shown an advertisement the first time they logged in to the app during four 24-hour periods [17]. The advertisement contained text encouraging users to click through to complete an anonymous Web-based survey on the health of gay and bisexual men in the United Kingdom. Participants were told that completing the survey would enter them in a lottery for a chance to earn £50 (US $67). At the end of the recruitment period, 1410 users had clicked through the advertisement and 202 users completed the survey, representing a cooperation rate of 14.33%. All protocols were approved via institutional review board prior to data collection.

### Survey Measures

#### Willingness to Use Mobile Phone Apps for HIV Prevention

Respondents were asked, “How willing would you be to use an app to remind yourself when to get tested for HIV?” The wording of this potential intervention was selected given its alignment with the goals of primary prevention of HIV infection [18]. Using a 5-point Likert scale, response options for this item ranged from very willing to very unwilling. For analytical purposes, these categories were collapsed into willing, undecided, and unwilling.

#### Geosocial-Networking Mobile Phone App Use

Given that previous research has shown that many individuals who use health-related apps download them and then discontinue using them [8], all respondents were asked, “In the past 12 months, how many times have you deleted and re-downloaded Grindr?” with the aim of understanding consistency of use of geosocial-networking apps to connect with other MSM. Response options for this item included 8 or more times, 6 or 7 times, 4 or 5 times, 2 or 3 times, 1 time, and 0 times.

#### Recent Sexual Behaviors

All respondents were asked to report the total number of partners with whom they had insertive and receptive anal intercourse with and without a condom in the preceding 3 months. These counts were then dichotomized (0 partners vs 1 or more partners).

#### Recent Substance Use Behaviors

Respondents were asked to select from a list of substances to indicate any use in the preceding 3 months, included alcohol (≥5 drinks in 1 sitting), cocaine, ecstasy, gamma-hydroxybutyric acid or gamma-butyrolactone (GHB/GBL), inhalant nitrites, ketamine, lysergic acid diethylamide (LSD), marijuana, and methamphetamine. Each of these substances was considered individually and a composite variable was created for club drug use (eg, use of ecstasy, GHB/GBL, ketamine, and/or LSD).

#### Demographic Variables

Respondents reported their age, sexual orientation, ethnic group membership, employment status, relationship status, and HIV status. Age was measured continuously in years and categorized as aged 18 to 24 years, 25 to 30 years, 31 to 40 years, 41 to 50 years, and 51 years and older. Sexual orientation was categorized as gay, bisexual, straight, and other. Ethnic group membership was categorized as white, black, Asian, and mixed/multiple ethnic groups/other. Employment status was categorized as employed, unemployed, retired, or student. Relationship status was categorized as being in a primary relationship with another man or not. HIV status was assessed based on self-report as negative or positive.

### Statistical Analyses

Broadcast advertisements yielded an overall sample of 202 respondents within 96 hours. A total of 10 individuals who did not report their HIV status and 23 who self-reported as HIV-positive were excluded, restricting the analytical sample to 169 respondents. Descriptive statistics were calculated for all variables. Differences in willingness to use apps for HIV prevention purposes by demographic variables, recent substance use, and categorical measures of recent sexual behaviors were assessed using chi-square tests of independence. Significance was determined as *P*<.05.

## Results

### Sample Demographics

The average age of the sample was 36.7 (SD 11.5) years. A majority (165/169, 97.9%) self-identified as gay or bisexual. Almost three-fourths (122/169, 72.4%) identified their ethnic group as white or white British. A large percentage (144/169, 85.5%) were either currently employed or enrolled in school. Approximately one-fifth (32/169, 18.8%) reported currently being in a relationship with another man.

### Recent Sexual Behaviors

Three-quarters of respondents (127/169, 75.2%) engaged in insertive anal intercourse in the preceding 3 months. Among respondents who had engaged in insertive anal intercourse, 59.9% (76/127) engaged in condomless insertive anal intercourse with 1 or more partners. Almost two-thirds of respondents (110/169, 64.9%) engaged in receptive anal intercourse in the preceding 3 months. Among respondents who had engaged in receptive anal intercourse, 57.3% (63/110) engaged in condomless receptive anal intercourse with 1 or more partners. About two-fifths of respondents (72/169, 42.6%) had engaged in a group sex event in the preceding 3 months.

### Recent Substance Use Behaviors

Two-fifths of respondents (65/169, 38.6%) reported having had 5 or more drinks containing alcohol in 1 sitting in the preceding 3 months. With regard to other substances, 36.6% (62/169) reported using inhalant nitrites, 23% (39/169) reported using club drugs, 19.8% (33/169) reported using marijuana, 14.4% (24/169) reported using cocaine or crack cocaine, and 13.9% (23/169) reported using methamphetamine in the preceding 3 months.

### Geosocial-Networking Mobile Phone App Use

About half of respondents (82/169, 48.5%) had not deleted Grindr in the preceding year. Among those who had deleted and re-downloaded Grindr in the preceding year, 69.2% (57/82) had deleted and re-downloaded the app 3 or fewer times.

### Willingness to Use Mobile Phone Apps for HIV Prevention

Descriptive statistics are displayed in Table 1. Among the sample, 63.9% (108/169) were willing to use an app to remind them to get tested for HIV, 24.9% (42/169) were undecided, and 11.2% (19/169) were unwilling.

Exploratory analyses examining differences in self-reported willingness to use an app for HIV prevention by demographic characteristics (eg, age, ethnic group membership) and recent sexual (eg, engagement in condomless insertive anal intercourse) and substance use (eg, alcohol use, club drug use) behaviors are shown in Table 1. Respondents who reported binge drinking (*P*=.03) and club drug use (*P*=.04) more frequently reported more willingness to use an app for HIV testing reminders than their nonusing counterparts.

**Table 1:** Willingness to use an app for HIV testing reminders among HIV-negative men who have sex with men recruited from a popular social-networking app in London (n=169).

Demographic/behavioral characteristic		Frequency, n (%)	Rated very willing/willing, n (%)	*P* value
**Age, years**				.69
	18 to 24	30 (16.8)	18 (69.3)	
	25 to 30	46 (25.7)	24 (54.6)	
	31 to 40	55 (30.7)	36 (70.6)	
	41 to 50	30 (16.8)	17 (56.6)	
	51 and older	18 (10.1)	13 (72.2)	
**Sexual orientation**				.80
	Gay	156 (87.2)	92 (62.4)	
	Bisexual	19 (10.6)	13 (76.5)	
	Other	4 (2.2)	2 (66.7)	
**Ethnic group membership**				.99
	White/white British	128 (71.5)	80 (65.6)	
	Black/black British	10 (5.6)	6 (66.6)	
	Asian/Asian British	14 (7.8)	6 (54.6)	
	Mixed/multiple ethnic groups/other	25 (14.0)	14 (56.0)	
**Employment status**				.33
	Employed	133 (74.3)	80 (62.6)	
	Unemployed	17 (9.5)	9 (60.0)	
	Student	23 (12.8)	13 (75.0)	
	Retired	5 (2.8)	5 (100.0)	
**Primary relationship with man**				.97
	Yes	32 (17.9)	18 (62.0)	
	No	147 (82.1)	90 (64.2)	
**Recent sexual behaviors**				
	Condomless receptive anal intercourse	62 (34.6)	39 (69.6)	.09
	Condomless insertive anal intercourse	76 (42.5)	47 (65.8)	.50
**Recent substance use**				
	Alcohol (≥5 drinks in a row)	71 (397)	48 (71.6)	.03
	Cocaine	26 (14.5)	15 (62.5)	.18
	Club drugs	40 (22.3)	30 (78.9)	.04
	Inhalant nitrites	63 (35.2)	43 (70.5)	.69
	Marijuana	38 (21.2)	24 (66.7)	.94
	Methamphetamine	21 (11.7)	18 (90.0)	.12

## Discussion

### Principal Findings

This is the first study to examine the acceptability of the use of mobile phone apps for HIV testing reminders among a sample of MSM in London who use geosocial-networking apps to meet other MSM. Overall, respondents reported a willingness to use an app to remind them to be periodically tested for HIV (108/169, or 63.9%, rated themselves as willing or very willing). Our findings are similar to those observed among a sample of young MSM in Southern California, where about 70.0% (137/195) were willing to participate in an HIV prevention program delivered via an app [19]. In addition, we found no significant differences in willingness to use these apps based on demographic characteristics or sexual behaviors. The lack of significant variations in willingness to use these apps across most subgroups in this sample of MSM suggests that these intervention tools can be implemented in diverse populations of MSM living in London. However, the presence of significant differences among MSM who reported binge drinking and club drug use may suggest a need for targeted prevention-related apps for these populations so that they can manage their HIV-related risks and substance use-related risks concurrently.

Given the high prevalence of inconsistent use of geosocial-networking apps to meet sexual partners in the preceding 12 months (where 87/169, or 51.5%, reported deleting and re-downloading the app at least once), the implementation of these interventions should include strategies to encourage consistency of use. Recent formative qualitative research has aimed at assessing factors associated with consistency of use. Among samples of HIV-negative MSM in Miami and Minneapolis, reliability, ease of use, and frequency of updates were cited as factors associated with whether an individual would keep and continue to use an app over time while poor performance and functionality and lack of use were primary reasons why MSM would delete an app from their phone [20]. The item used in this study examines consistency of use of social-networking apps, and this may not reflect potential consistency of use of health-related apps or HIV prevention-related apps. However, it is possible that HIV prevention-related features of app-based interventions may be more consistently used if integrated into an existing social-networking platform.

Future research is needed to identify and test potential apps to be used for HIV testing reminders among MSM who use geosocial-networking apps to meet sexual partners, particularly in contexts other than the United States, given that this is where most of the previous research has been conducted [21,22]. However, it is possible that the features of apps desired by MSM in the United States may be similar to those desired by MSM elsewhere. Previous research with HIV-negative MSM has suggested that apps for HIV prevention should encourage regular HIV testing by providing feedback on test reminders, tailored testing interval recommendations, and HIV test site-locating features [20]. In addition, these apps should be designed to be discrete in nature, protect privacy, and not necessarily appear overtly to be related to HIV prevention. Should another individual, for example, see an app explicitly for HIV prevention, it could expose an individual to HIV-related stigma (if others assume they are HIV-positive) and discourage them from using these types of apps.

### Limitations

These findings are not without limitations. These findings are derived from data collected as part of a convenience sample of MSM from a single popular geosocial-networking app in a single metropolitan center in Western Europe. As such, these findings are likely not generalizable to broader populations of MSM on other apps or outside of these apps. These findings are also likely biased by some degree of self-selection, where those who are willing to engage with communication regarding HIV prevention via apps may have been more likely to participate. In addition, our measures of risk behaviors (eg, sexual and substance use behaviors) are crude, and there may be some misclassification with regard to behavioral risk. Future research should use more nuanced measures of these behaviors (eg, number of events of condomless receptive anal intercourse with a serodiscordant or unknown HIV status partner in a recall period, number of times using methamphetamine in a recall period) to further our understanding of the behavioral risk profiles of MSM who are willing to use apps for HIV prevention.

### Conclusion

The majority of respondents in this sample of MSM who use apps to meet other MSM reported willingness to use an app to remind them to be tested for HIV infections. Apps to be used for HIV prevention interventions, such as those aimed at increasing HIV testing rates among MSM (particularly those who are already using apps to meet other MSM), should be developed, evaluated, and implemented.

## Abbreviations

GBL: gamma-butyrolactone
GHB: gamma-hydroxybutyric acid
GPS: Global Positioning System
HIV: human immunodeficiency virus
LSD: lysergic acid diethylamide
MSM: men who have sex with men
STI: sexually transmitted infection
